# Structural changes of cerebellum and brainstem in migraine without aura

**DOI:** 10.1186/s10194-019-1045-5

**Published:** 2019-09-02

**Authors:** Zhaoxia Qin, Xin-Wei He, Jilei Zhang, Shuai Xu, Ge-Fei Li, Jingjing Su, Yan-Hui Shi, Shiyu Ban, Yue Hu, Yi-Sheng Liu, Mei-Ting Zhuang, Rong Zhao, Xiao-Lei Shen, Jianqi Li, Jian-Ren Liu, Xiaoxia Du

**Affiliations:** 1 Shanghai Key Laboratory of Magnetic Resonance and Department of Physics, School of Physics and Electronic Science, East China Normal University, 3663 North Zhong-Shan Road, 200062 Shanghai, People’s Republic of China; 2 Department of Neurology and Jiuyuan Municipal Stroke Center, Shanghai Ninth People’s Hospital, Shanghai Jiao Tong University School of Medicine, 639 Zhizaoju Road, 200011 Shanghai, People’s Republic of China; 3 Clinical Research Center, Shanghai Jiao Tong University School of Medicine, Shanghai, 200011 China

**Keywords:** Migraine, cerebellum, brainstem, diffusion tensor images, voxel-based morphometry

## Abstract

**Background:**

Increasing evidence has suggested that the cerebellum is associated with pain and migraine. In addition, the descending pain system of the brainstem is the major site of trigeminal pain processing and modulation and has been discussed as a main player in the pathophysiology of migraine. Cerebellar and brainstem structural changes associated with migraineurs remain to be further investigated.

**Methods:**

Voxel-based morphometry (VBM) (50 controls, 50 migraineurs without aura (MWoAs)) and diffusion tensor imaging (DTI) (46 controls, 46 MWoAs) were used to assess cerebellum and brainstem anatomical alterations associated with MWoAs. We utilized a spatially unbiased infratentorial template toolbox (SUIT) to perform cerebellum and brainstem optimized VBM and DTI analysis. We extracted the average diffusion values from a probabilistic cerebellar white matter atlas to investigate whether MWoAs exhibited microstructure alterations in the cerebellar peduncle tracts.

**Results:**

MWoAs showed decreased fractional anisotropy (FA) in the vermis VI extending to the bilateral lobules V and VI of the cerebellum. We also found higher axial diffusivity (AD), mean diffusivity (MD), and radial diffusivity (RD) in the right inferior cerebellum peduncle tract in MWoAs. MWoAs exhibited both reduced gray matter volume and increased AD, MD and RD in the spinal trigeminal nucleus (SpV).

**Conclusion:**

MWoAs exhibited microstructural changes in the cerebellum and the local brainstem. These structural differences might contribute to dysfunction of the transmission and modulation of noxious information, trigeminal nociception, and conduction and integration of multimodal information in MWoAs. These findings further suggest involvement of the cerebellum and the brainstem in the pathology of migraine without aura.

**Electronic supplementary material:**

The online version of this article (10.1186/s10194-019-1045-5) contains supplementary material, which is available to authorized users.

## Introduction

Migraine is a common and disabling neurological disorder that manifests as moderate to severe intensity headaches generally combined with nausea, vomiting, and hypersensitivity to visual, auditory, and olfactory stimuli [1, 2]. Migraine, which affects nearly 1 billion people worldwide, especially women, has become a major public health concern [3, 4]. Therefore, approaches to better understand the pathophysiology of migraine may aid in developing better diagnostic and treatment plans for these patients.

In the past decade, increasing evidence has suggested that the cerebellum is associated with pain and migraine [5–9]. The cerebellum is a highly-organized brain area located in the hindbrain dorsal to the brainstem, which has been proposed to be related to the processing of sensorimotor, affective and cognitive information [10–12]. Recent studies also demonstrated the involvement of the cerebellum in human nociception [13], and even suggest a modulating role in pain perception [14]. Several previous studies have reported that migraineurs exhibit functional abnormalities in the cerebellum [8, 15, 16]. Migraine research also found an increased prevalence of ischemic lesions particularly in the cerebellar posterior lobe of migraineurs [17]. Furthermore, the cerebellum exhibited remarkably high concentrations of calcitonin gene-related peptide [18],a strong vasodilator which is a target peptide for migraine treatment [19]. Previous study found the volumes of the cerebellum and brainstem were smaller in chronic migraine compared to healthy controls[20]. Thus, there is ample evidence that the cerebellum plays significant role in the pathology of migraine. However, cerebellar anatomy alterations associated with migraineurs need further investigated. Using brainstem and cerebellar specific analysis techniques to make more accurate segmentation and spatial normalization is essential [21].

The cerebellar peduncles are major white matter tracts that communicate information among the cerebellum, the cerebral cortex, and the spinal cord. A recent study found that migraine patients have less superior cerebellar peduncle volume and that migraineurs with lower heat pain thresholds have smaller superior cerebellar peduncles [22]. These studies suggest that altered structure volume of the cerebellar peduncles is associated with migraine, while the diffusion characteristics of cerebellar peduncle tracts require further investigation. The white matter lesions of the cerebellar peduncle tracts may disconnect these relay stations in the cerebrocerebellar circuitry, eventually leading to this complex disorder. Thus, the diffusion characteristics of the cerebellar peduncles in migraineurs should be further investigated.

The descending pain system of the brainstem is the major site of trigeminal pain processing and modulation and has been discussed as a main player in the pathophysiology of migraine. Human brain imaging studies have shown that, during a migraine attack, activity increases in brainstem nuclei such as spinal trigeminal nucleus (SpV), the dorsal pons and the midbrain periaqueductal gray matter [23, 24]. A number of studies have also reported altered sensitivity to somatosensory stimuli in the brainstem of migraine patients[2]. Functional connectivity analysis showed enhanced functional coupling between the hypothalamus and the SpV during the preictal phase compared with during the interictal phase [25]. Several brain morphology studies have shown that migraine is associated with decreased gray matter volume (GMV) and diffusivity abnormalities in areas involved in pain processing in the brainstem [26, 27]. Brainstem structural alterations, which might underlie the functional pathology of migraines, should be further investigated using advanced techniques.

Diedrichsen presents the Spatially Unbiased Infra-tentorial (SUIT) template of human brainstem and cerebellum, it allows for an improved voxel-by-voxel normalization for functional MRI analysis [21]. In this study, we used voxel-based morphometry of T1-weighted anatomical and diffusion weighted images, in combination with the brainstem and cerebellum template from Diedrichsen [21] to test the following hypotheses 1) migraineurs without aura (MWoAs) may exhibited altered GMV at the cerebellum, brainstem; 2) MWoAs may displays altered diffusion characters at cerebellum, cerebellar peduncles tracts and brainstem; 3) these structural alterations may associated with disease severity, such as pain intensity, disease duration, attack frequency, Migraine Disability Assessment Scale (MIDAS) and Headache Impact Test (HIT-6) scores.

## Materials and methods

### Subjects

Patients were recruited from the outpatient clinic of the Department of Neurology at Shanghai Ninth People’s Hospital and diagnosed with migraine without aura by a neurologist based on the International Classification of Headache Disorders 3rd edition criteria [1]. We compared voxel-based morphometric of T1-weighted anatomical images between 50 MWoAs (mean ± SD age=38.7±11.2 years) and 50 age- and gender-matched healthy controls (HCs) (mean ± SD age = 39.5±11.3 years). Because four MWoAs did not complete the diffusion tensor imaging (DTI) scans, we compared the diffusion tensor images of 46 MWoAs (38.8±11.6) and 46 HCs (39.0±11.2). We obtained demographic and clinical data, including age, sex, disease duration, attack frequency (times/month), attack duration (hours) and pain intensity of migraine attacks assessed by a visual analogue scale (VAS)[28], of the MWoAs from the neurologist. Patients also completed the MIDAS[29] and HIT-6[30] to obtain an accurate assessment of their headache-related disability. In this study, all MWoAs were scanned during an interictal period, that is, at least no migraine attacks had occurred 48 hours before or 24 hours after MRI scanning. Migraineurs reported that they did not take preventive medication and did not have chronic migraine. The HCs had no headaches or chronic pain disorders in the past year and no neurological, psychiatric diseases, metabolic disease (e. g. diabetes mellitus) or cardiovascular disease. Moreover, the immediate family members of the HCs did not suffer from migraine or other headaches. Participants were all right handed and did not report drug abuse. The demographic and clinical data are provided in Table 1.

**Table 1:** Demographic data and clinical scores of the migraine and control groups.

	Migraine group	Control group	
	(Mean ± SD)	(Mean ± SD)	*P* value
N	50	50	1
Sex (male)	30%	30%	1
Age (years)	38.7±11.2	39.5±11.3	0.71
Disease duration (years)	8.6±6.2	-	-
Attack duration (hours)	17.3±19.5	-	-
Attack frequency (times/months)	3.3±2.8	-	-
Pain intensity VAS	7.1±1.9	-	-
MIDAS	19.6±25.7	-	-
HIT-6	60.5±11.7	-	-

*VAS* Visual analogue scale, *MIDAS* Migraine disability assessment scale, *HIT-6* Headache impact test, - No data

### MRI acquisition

Magnetic resonance imaging (MRI) was performed on a 3.0 Tesla Siemens Trio Tim system equipped with a 12-channel head coil at the Shanghai Key Laboratory of Magnetic Resonance (East China Normal University, Shanghai, China). The subjects were instructed not to move during the scans, and a Siemens dedicated filler was used to prevent head movement. High-resolution T1-weighted anatomical images were acquired by using a 3-dimensional magnetization-prepared rapid-acquisition gradient-echo pulse sequence (repetition time, 2530 ms; echo time, 2.34 ms; inversion time, 1100 ms; flip angle, 7°; number of slices, 192; sagittal orientation; field of view, 256×256 mm^2^; and voxel size, 1 × 1 × 1 mm^3^). The DTI acquisition utilized a single-shot spin-echo planar imaging sequence in the contiguous axial plane with the following parameters: repetition time, 8900 ms; echo time, 86 ms; b-values, 0 and 1000 s/mm^2^; diffusion direction, 64; number of slices, 70; field of view, 256 × 256 mm^2^; and voxel size, 2 × 2 × 2 mm^3^.

### VBM analysis

The cerebellum and brainstem optimized voxel-based analysis was performed using the spatially unbiased infratentorial template (SUIT) [21] toolbox implemented in Statistical Parametric Mapping software, version 12 (spm12, http://www.fil.ion.ucl.ac.uk/spm). We cropped and masked each T1 image before reslicing the image into the SUIT space so that no supratentorial gray matter (GM) could bias the results. After visually checking for data artifacts and setting the image origin at the anterior commissure of each subject, we isolated the cerebellum and brainstem structures from the surrounding tissues. To exclude any tissue outside the brainstem or the cerebellum, we hand-corrected the isolated maps using MRIcron software (http://people.cas.sc.edu/rorden/mricron). The subsequent spatial normalization and reslicing yielded cerebellar and brainstem “maps” of GM probabilities modulated by volume changes due to normalization using the ‘preserve’ option. The maps were resliced into the Montreal Neurological Institute (MNI) space, and a small smoothing kernel (3 mm full width-at-half-maximum) was used to maintain spatial accuracy in small brainstem sites. Then, we conducted a voxelwise two-sample T test with sex and age as covariates to compare GMV between the two groups. The threshold was p<0.001, two-tailed at the voxel level, and family wise error (FWE)-corrected to p<0.05 at the cluster level. We used Duvernoy’s Atlas of the Human Brainstem and Cerebellum [31] to identify the locations of significantly different clusters overlaid onto the SUIT brainstem template.

### DTI analysis

DTI data were preprocessed and analyzed using the FMRIB's Diffusion Toolbox in FSL software (FMRIB Software Library, http://www.fmrib.ox.ac.uk/fsl). The original data were corrected for the effects of head movement and eddy currents using the eddy correct command by applying affine alignment of each diffusion-weighted image to the first b = 0 image. The diffusion tensor was calculated from the images using a linear model, and then, fractional anisotropy (FA), axial diffusivity (AD), mean diffusivity (MD) and radial diffusivity (RD) whole-brain maps were derived. These images were then coregistered to each individual subject’s T1-weighted image, and then, the cerebellum and brainstem were isolated, spatially normalized and resliced to the SUIT template using the parameters derived from the aforementioned T1-weighted SUIT analysis. This process resulted in cerebellum and brainstem maps of diffusion values, spatially normalized in the MNI space with raw intensities preserved (nonmodulated). The images were smoothed (3 mm full-width-at-half-maximum), and we used SPM software to conduct a voxelwise two-sample T test with sex and age as covariates to compare the diffusion characteristics between the two groups. The thresholds were p<0.001, two-tailed at the voxel level, and FWE-corrected to p<0.05 at the cluster level. We used Duvernoy’s Atlas of the Human Brainstem and Cerebellum [31] to identify the locations of significantly different clusters overlaid onto the SUIT brainstem template.

### Cerebellum tract analysis

We extracted the average diffusion values from a probabilistic cerebellar white matter atlas[32] at a 90% threshold, which included the superior, middle and inferior cerebellar peduncles (Additional file 1: Figure S1). We used a two-sample T test with sex and age as covariates to determine if there was a significant difference in diffusion characteristics between the MWoAs and the HCs in these tracts. Significant differences were determined according to p<0.0083 (Bonferroni corrected for multiple comparisons, p<0.05).

### Correlation analysis

Individual altered mean GMVs and diffusion characteristics for the surviving clusters and cerebellar tracts of the MWoAs were extracted for Spearson’s correlation with the clinical data, including disease duration, attack frequency, attack duration, and VAS, MIDAS, and HIT-6 scores. Significant correlations were determined according to p<0.0083 (Bonferroni corrected for multiple comparisons, p<0.05).

## Results

### Demographic and clinical characteristics

The demographic and clinical data of the MWoA and control groups are presented in Table 1. There was no significant difference in age (two-sample T test; p>0.05).

### Gray matter volume changes (VBM)

A comparison of GMVs revealed a GMV decrease in the SpV in migraineurs relative to controls, as shown in Figure 3 (p<0.001 at the voxel level, FWE-corrected p=0.067 at the cluster level). The peak MNI coordinates were (4, -45, -65).

### Changes in diffusion characteristics (DTI)

**Fig. 1:**
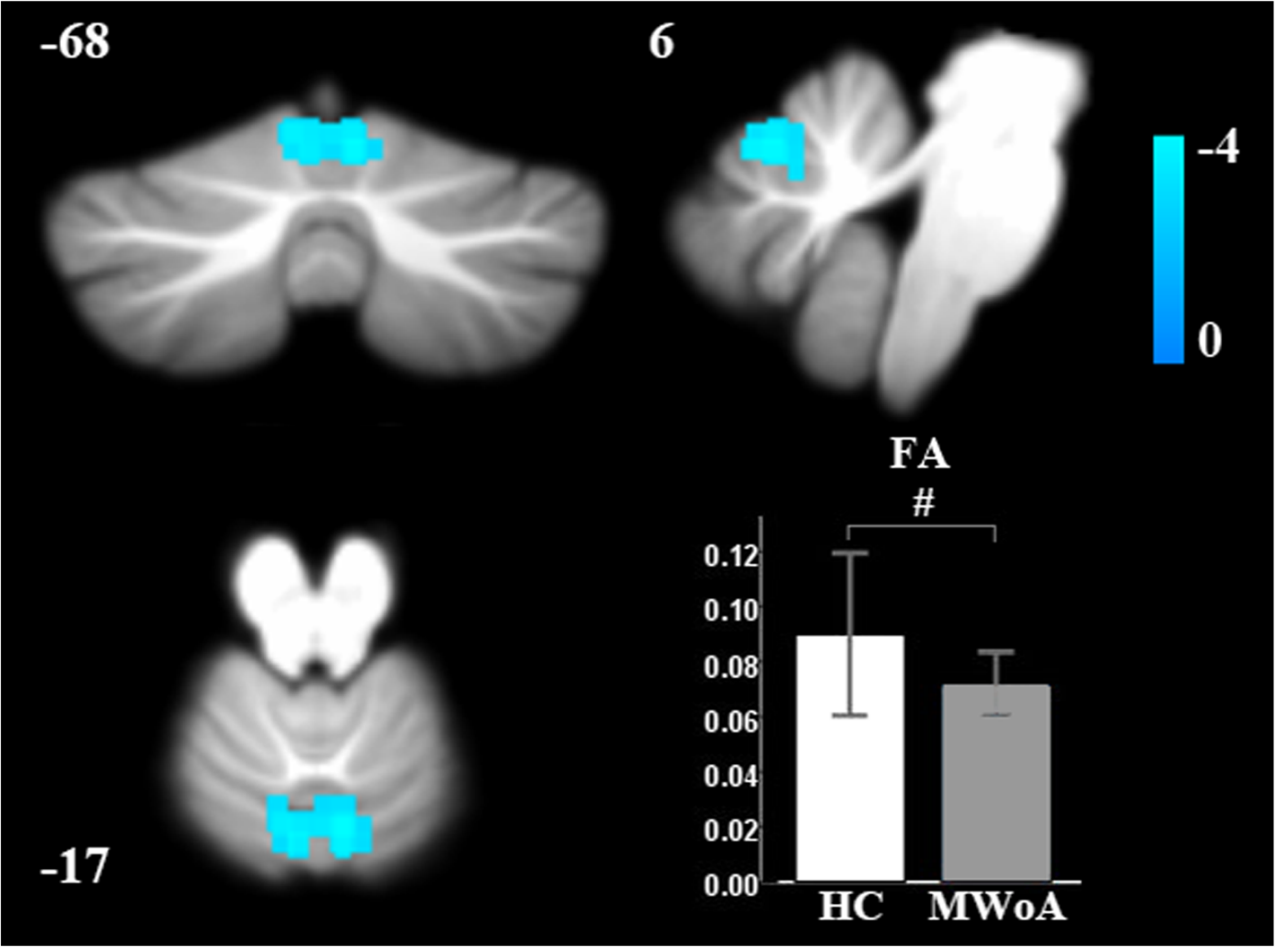
Brain regions with significantly decreased fractional anisotropy (FA) of MWoAs relative to healthy controls. ^#^ Statistical significance of p<0.001 at the voxel level, FWE-corrected p<0.05 at the cluster level.

**Fig. 2:**
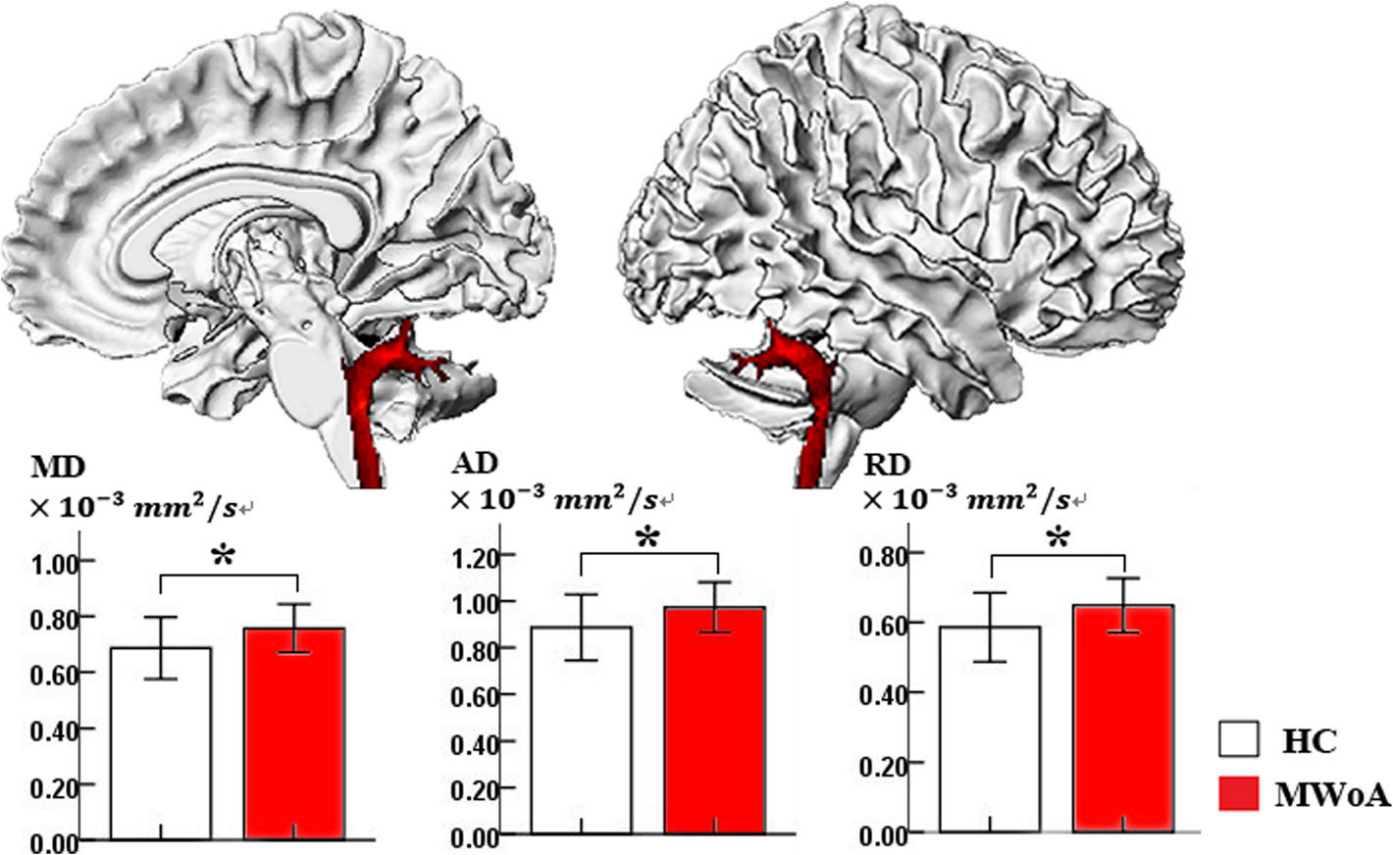
Sagittal view of the right inferior cerebellar peduncle with significantly increased mean diffusivity (MD), axial diffusivity (AD), and radial diffusivity (RD) in MWoAs relative to healthy controls; *Statistical significance was p<0.0083 (Bonferroni corrected for multiple comparisons p<0.05).

**Fig. 3:**
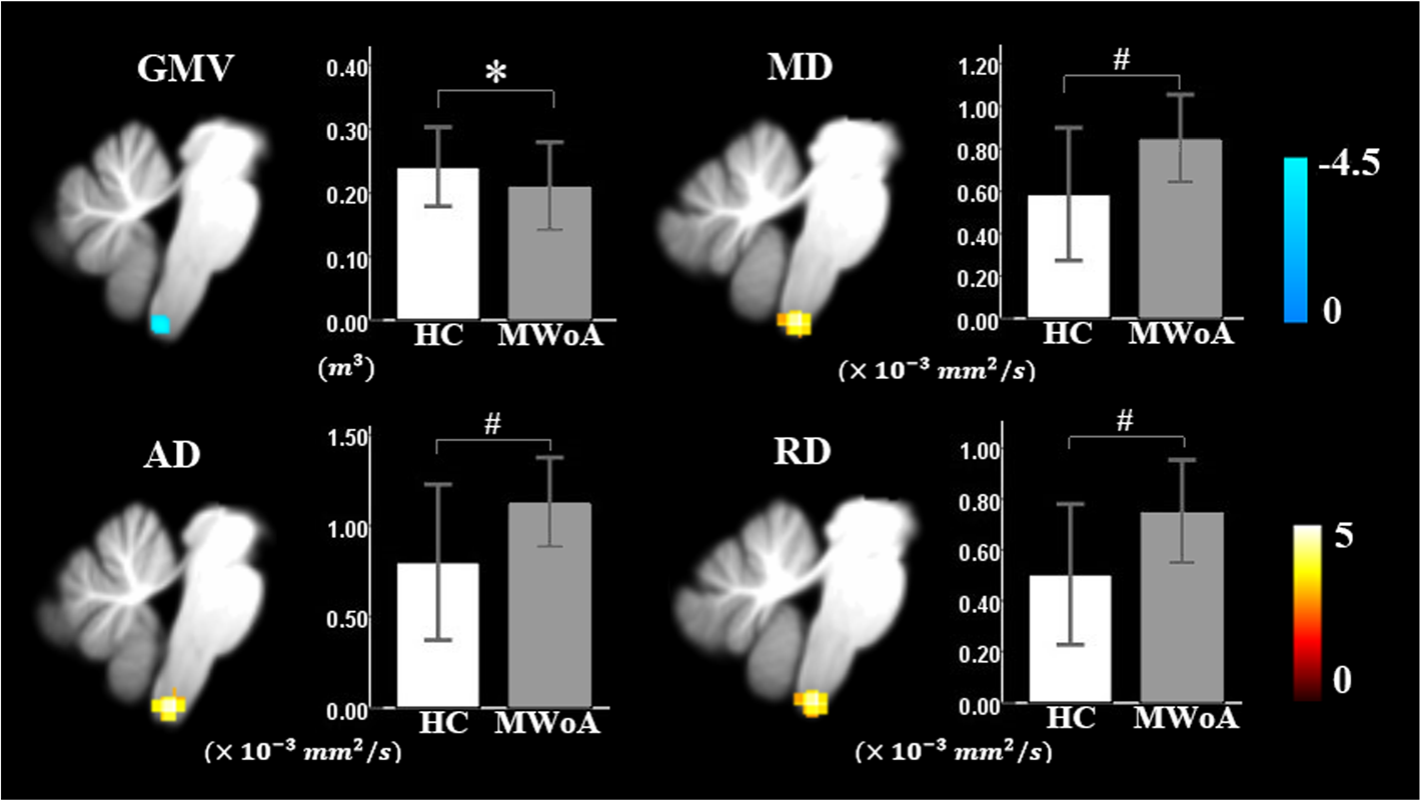
Sagittal view of the spinal trigeminal nucleus (SpV) with significant differences in gray matter volume and diffusion characteristics between MWoAs and healthy controls. * Statistical significance of p<0.001 at the voxel level, FWE-corrected p<0.067 at the cluster level; # Statistical significance of p<0.001 at the voxel level, FWE-corrected p<0.05 at the cluster level.

Compared with controls, MWoAs had decreased FA at the vermis VI extending to the bilateral lobule V and the bilateral lobule VI of the cerebellum (Table 2, Fig. 1). Cerebellar tract analysis revealed that MWoAs had higher AD, MD and RD in the right inferior cerebellum peduncle (ICP) (Table 3, Fig. 2). MWoAs had higher AD, MD, and RD in the region of the SpV (Table 2, Fig. 3).

**Table 2:** Brain regions with significant differences in diffusion characteristics between MWoAs and healthy controls.

	Cluster size	Peak T value	MNI coordinates			Cluster level
			x	y	z	*p*_*FWE* − *corr*_
FA: (Migraine group < Control group)						
Vermis VI extending to the bilateral lobule V and bilateral lobule VI	163	4.18	6	-68	-17	0.002
AD: (Migraine group > Control group)						
SpV	123	4.72	-2	-42	-63	0.026
MD: (Migraine group > Control group)						
SpV	170	5.00	-4	-42	-65	0.006
RD: (Migraine group > Control group)						
SpV	173	5.08	-4	-42	-65	0.004

The results were assigned thresholds at p<0.001 (voxel level) and FWE-corrected to p<0.05 at the cluster level. *AD* Axial diffusivity, *MD* Mean diffusivity, *RD* Radial diffusivity, *FA* Fractional anisotropy, *SpV* Spinal trigeminal nucleus

**Table 3:** Diffusion characteristics for 3 major cerebellar tracts in the MWoA and healthy controls.

	L-SCP	R-SCP	L-MCP	R-MCP	L-ICP	R-ICP
AD						
HC	0.92±0.14	0.91±0.14	0.83±0.12	0.86±0.12	0.90±0.14	0.89±0.14
MWoA	0.94±0.10	0.96±0.10	0.81±0.08	0.84±0.08	0.96±0.10	0.97±0.11
P value	0.590	0.069	0.324	0.462	0.045	0.002*
MD						
HC	0.63±0.11	0.62±0.10	0.57±0.14	0.59±0.13	0.70±0.12	0.69±0.11
MWoA	0.63±0.07	0.66±0.08	0.52±0.05	0.55±0.05	0.75±0.08	0.76±0.09
P value	0.997	0.072	0.032	0.044	0.057	0.001*
RD						
HC	0.48±0.10	0.48±0.09	0.44±0.15	0.45±0.15	0.60±0.11	0.59±0.10
MWoA	0.47±0.06	0.51±0.07	0.38±0.04	0.40±0.04	0.64±0.07	0.65±0.08
P value	0.694	0.094	0.013	0.015	0.079	0.001*
FA						
HC	0.30±0.06	0.30±0.05	0.37±0.12	0.37±0.12	0.22±0.05	0.21±0.05
MWoA	0.31±0.04	0.31±0.04	0.41±0.05	0.41±0.05	0.23±0.03	0.23±0.03
P value	0.157	0.162	0.012	0.011	0.094	0.125

*AD* Axial diffusivity, *MD* Mean diffusivity, *RD* Radial diffusivity, *FA* Fractional anisotropy, *SCP* Superior cerebellar peduncle, *MCP* Middle cerebellar peduncle, *ICP* Inferior cerebellar peduncle, * Statistical significance was set at p<0.0083, (Bonferroni corrected p<0.05). The MD, RD, and AD metrics are shown as 10^-3^, and FA is shown as the actual value. Note that only the right inferior cerebellar peduncle displayed significant increases in MD, RD, and AD compared with controls.

### Correlation with clinical scores

Altered GMVs and diffusion characteristics were not significantly correlated with MWoA clinical data (Table 4).

**Table 4:** Clinical correlations with altered GMVs and diffusion characteristics of patients with MWoA.

	Disease duration (years)	Duration (hours)	Frequency (times/ months)	Pain intensity VAS	MIDAS	HIT-6
Decreased GMVs at SpV						
r	0.119	0.169	-0.249	0.044	-0.221	0.038
P value	0.432	0.261	0.096	0.773	0.140	0.800
Decreased FA at Vermis VI extending to the bilateral lobule V and bilateral lobule VI						
r	-0.047	-0.250	0.347	0.006	0.190	0.110
P value	0.759	0.094	0.018	0.970	0.206	0.465
Increased AD at R-ICP						
r	-0.058	-0.125	0.160	0.218	0.106	0.120
P value	0.703	0.407	0.289	0.146	0.485	0.425
increased MD at R-ICP						
r	-0.105	-0.072	0.139	0.147	0.064	0.050
P value	0.486	0.633	0.355	0.330	0.671	0.741
increased RD at R-ICP						
r	-0.155	-0.051	0.123	0.114	0.065	0.033
P value	0.305	0.734	0.416	0.449	0.670	0.827
increased AD at SpV						
r	-0.246	0.034	0.107	-0.023	-0.050	-0.135
P value	0.099	0.823	0.479	0.881	0.740	0.370
increased MD at SpV						
r	-0.234	0.053	0.105	-0.036	-0.038	-0.165
P value	0.118	0.728	0.486	0.814	0.803	0.273
increased RD at SpV						
r	-0.250	0.087	0.106	-0.044	-0.041	-0.162
P value	0.094	0.566	0.482	0.770	0.786	0.283

*VAS* Visual analogue scale, *MIDAS* Migraine disability assessment scale, *HIT-6* Headache impact test; Frequency: the average number of attacks per month in the last three month; *AD* Axial diffusivity, *MD* Mean diffusivity, *RD* Radial diffusivity, *FA* Fractional anisotropy, *SCP* Superior cerebellar peduncle, *MCP* Middle cerebellar peduncle, *ICP* Inferior cerebellar peduncle, *SpV* Spinal trigeminal nucleus, *r* Spearson’s rho; Significant correlations were determined according to p<0.0083 (Bonferroni corrected for multiple comparisons, p<0.05).

## Discussion

In the present study, we used the SUIT toolbox to explore alterations in GMVs and diffusion properties of the cerebellum and the brainstem associated with MWoAs. We found decreased FA properties in the vermis VI extending to the bilateral lobule V and the bilateral lobule VI of the cerebellum. Higher AD, MD, and RD were found in the right inferior cerebellum peduncle tract in MWoAs. We also found both reduced GMV and increased AD, RD, and MD with regard to microstructural alterations that occurred in all divisions of the SpV. In migraine subjects, these GMV and diffusivity changes were not correlated with migraine frequency, duration or other clinical data, which may due to the limited number of patients. In the other side, we think that repeated, long-term headache attacks may induce adaptive or maladaptive changes, and such changes would be highly complex and nonlinear.

We found that MWoAs exhibited decreased FA in the vermis VI extending to the bilateral lobule V and the bilateral lobule VI compared with HCs. FA is a parameter that can be used to investigate anisotropy and is a marker of the change in shape of the diffusion ellipsoid, which is highly sensitive to microstructural alterations[33]. Our results showed that MWoA-associated cerebellar microstructure alterations occurred at the vermis VI and at lobules V and VI. Stimulation of rat cerebellar cortex at posterior vermis (lobe VI), increases neural responses to a noxious visceral stimulus in and around the termination sites of nociceptive afferents in the spinal cord[34], which reflect cerebellar stimulation modulates nociception. In addition, a recent study found that trigeminal nociception is processed in the cerebellar lobules V and VI ipsilateral to the nociceptive stimulus[35]. An individual’s intensity and unpleasantness ratings are processed in the hemispheric lobule VI and extending to lobule V[35]. Importantly, lobule VI has been demonstrated to be involved in the integration of information across multiple modalities[36]. Patients’ microstructure alterations at the vermis VI and at lobules V and VI might have an impact on the trigeminal nociception and multimodal information integration, which could contribute to the pathology of MWoA.

We also found that MWoAs exhibited significantly higher AD, MD, and RD at the right ICP relative to HCs, which suggested that MWoAs exhibited altered microstructure of the ICP tract. The ICPs contain both afferent and efferent pathways and connect the medulla to the cerebellum[37]. The ICP carries many types of input and output fibers that are mainly related to integrating proprioceptive sensory input with motor vestibular functions, such as balance and posture maintenance[37]. The ICP connects the inferior olive to the cerebellum, and the inferior olive receives descending inputs from the parvicellular red nucleus, which in turn receives inputs from the primary motor cortex, the supplementary motor cortex, the premotor cortex, the primary somatosensory cortex, and the superior parietal lobe[38]. Based on these connections, the red nucleus-inferior olive system seems to convey motor and some sensory efferents to the cerebellum[38]. In addition, cerebellar anterior lobules receive sensorimotor input from the body through the ICPs [39]. Thus, the altered microstructure of the ICP tract suggests that MWoAs may exhibit dysfunctional conduction and integration of sensorimotor information at the medulla to cerebellum level, which could lead to higher-order dysfunction of cerebral cortex pain modulation and may be associated with the pathological mechanism of migraine without aura. In the future work, the microstructure of fiber tract connecting cerebellum to cerebral cortex remain to be further explored.

Previous study found the volumes of the cerebellum and brainstem were smaller in chronic migraineurs than healthy controls [20]. In our study, consistent with Marciszewski’s study [27], reduced GMV and increased MD were found in the SpV in MWoAs. In addition, we found increased AD and RD in the SpV, which suggested more subtle changes in tissue microstructure. These changes may result from loss of fiber tracts and disruption of the myelin sheath, increased membrane permeability, destruction of intracellular compartments, and glial alterations[40, 41]. The reduced GMV of the SpV in MWoAs may result from shrinkage or atrophy of neurons or glia or synaptic loss[42]. The SpV is a key structure in the brainstem involved in migraine pathophysiology, and it receives information from the trigeminal ganglion cells innervating the meninges and the cranial vasculature. Furthermore, the SpV is known to innervate several migraine-relevant structures, such as the thalamus and the hypothalamus [43]. Numerous human studies have suggested that the SpV plays an important role in the pathology of migraine. According to Stankewitz et al., the activity of the SpV in response to nociceptive stimulation predicted the time to the next headache [44]. Migraine subjects also showed greater amplification from the SpV to the posterior insula and the hypothalamus than controls [45]. Our results further supported that MWoAs have reduced GMV at the SpV and diffusion alterations of the SpV in three dimensions. These findings suggest that structural changes in the SpV may contribute to dysfunction of the SpV in transmitting noxious information from the cranial vessels and the meninges as well as atypical modulation of noxious inputs. Anatomical rat and human studies have described direct descending projections from the cerebral cortex to the SpV [46] and indirect projections arising in the cerebral cortex that project via the hypothalamus to the SpV [47]. Structural changes in the SpV might leading to higher-order dysfunction of cerebral cortex pain modulation.

Although our research revealed that MWoAs exhibit anatomical changes at the brainstem, the cerebellum and the cerebellar peduncles, the current study had several limitations. First, we examined patients only in the interictal phase, and therefore, brain structure in the ictal phase must also be explored. Second, we focused on anatomical changes in MWoAs but did not examine migraineurs with aura and chronic migraineurs. In a future study, we will evaluate the structural abnormalities of the brainstem and cerebellar areas of migraineurs with aura and chronic migraineurs during the ictal phase.

## Conclusion

In the present study, we found decreased FA in the vermis VI extending to the bilateral lobules V and VI of the cerebellum, which may contribute to the dysfunction of trigeminal nociception and multimodal information integration in MWoAs. We also found higher AD, MD, and RD in the right inferior cerebellum peduncle tract, which suggests that MWoAs exhibit atypical conduction and integration of sensorimotor information at the medulla to cerebellum level. MWoAs exhibited both reduced GMV and increased AD, RD, and MD in the SpV, which further strengthens the evidence linking migraine without aura to the SpV. Structural changes in the SpV could contribute to the dysfunction of the SpV in transmitting and modulating noxious information from the cranial vessels and the meninges.

## Additional file

Additional file 1: **Figure S1.** The probabilistic cerebellar white matter atlas (Van Baarsen et al., 2016, including the superior, middle and inferior cerebellar peduncles, at a threshold of 90%). (TIF 1124 kb)

## Data Availability

The datasets generated during and/or analysed during the current study are available from the corresponding author on reasonable request.

## Abbreviations

AD: Axial diffusivity
DTI: Diffusion tensor imaging
FA: Fractional anisotropy
FWE: Familywise error
GM: Gray matter
GMV: Gray matter volume
HCs: Healthy controls
HIT-6: Headache Impact Test
ICP: Inferior cerebellum peduncle
MD: Mean diffusivity
MIDAS: Migraine Disability Assessment Scale
MNI: Montreal Neurological Institute
MRI: magnetic resonance imaging
MWoAs: Migraineurs without aura
RD: Radial diffusivity
SpV: Spinal trigeminal nucleus
SUIT: Spatially Unbiased Infra-tentorial
VAS: Visual analogue scale
VBM: Voxel based morphometry
