# Supplementary figures and images for: Structural changes of cerebellum and brainstem in migraine without aura
Source: J Headache Pain. 2019 Sep 2;20(1):93. doi: 10.1186/s10194-019-1045-5 (PMC6734280; doi:10.1186/s10194-019-1045-5)

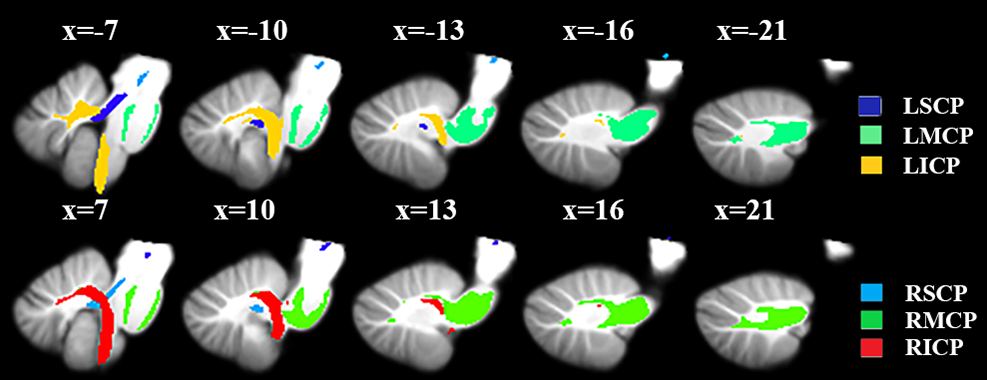

Supplement: Supplementary file 1 — Figure S1. The probabilistic cerebellar white matter atlas (Van Baarsen et al., 2016, including the superior, middle and inferior cerebellar peduncles, at a threshold of 90%). (TIF 1124 kb) [file 10194_2019_1045_MOESM1_ESM.tif]
